# The genus *Shirozuella* Sasaji (Coleoptera, Coccinellidae, Shirozuellini) from the Chinese mainland

**DOI:** 10.3897/zookeys.182.2430

**Published:** 2012-04-10

**Authors:** Xing-Min Wang, Feng Ge, Shun-Xiang Ren

**Affiliations:** 1 Institute of Zoology, Chinese Academy of Sciences, Beijing, 100101 China; 2 Engineering Research Center of Biological Control, Ministry of Education, South China Agricultural University, Guangzhou, 510642 China

**Keywords:** Coleoptera, Coccinellidae, Shirozuellini, *Shirozuella*, new species, China

## Abstract

The genus *Shirozuella* Sasaji, 1967 from the Chinese mainland is reviewed. Eight species are recognized, including four new species: *Shirozuella motuoensis* **sp. n.**, *Shirozuella tibetina* **sp. n.**, *Shirozuella unciforma* **sp. n.**, and *Shirozuella guoyuei* **sp. n.** Male genitalia of *Shirozuella parenthesis* Yu and *Shirozuella quadrimacularis* are described for the first time. All species are described and illustrated. A key and distribution map to the known species from the Chinese mainland are given.

## Introduction

The genus *Shirozuella* was established by Sasaji (1967) with *Shirozuella mirabilis* from Taiwan, China, as type species. It is a peculiar genus as follows: Clypeus distinctly expanded laterally, but antennal insertions not entirely hidden under the clypeus and distinctly visible in the lateral aspect, and labrum relatively long and exposed in front of the clypeus; antennae moderate in length with a distinct club; terminal maxillary palpomere very elongate, not distinctly broadening apically; underside of head capsule with deep furrows beside the mouth parts, for the reception of the retracted antennae. Based on these characteristics, Sasaji (1967) established the tribe Shirozuellini for reception of the genus *Shirozuella* and *Promecopharus* Sicard, 1910. Presently, Shirozuellini includes five genera (Miyatake 1994), *Shirozuella* Sasaji, *Promecopharus* Sicard, *Medamatento* Sasaji, 1989, *Ghanius* Ahmad, 1973, and *Sasajiella* Miyatake, 1994. Seago et al. (2011) listed *Guillermo* Ślipiński, 2007 and *Poorani* Ślipiński, 2007 as Shirozuellini (Coccinellinae) in a table. Although this was purported to be the original placement, Ślipiński (2007) had actually placed these genera in Coccidulini. Regardless, nothing in these recent analyses provided a justification for the inclusion of *Guillermo* and *Poorani* in Shirozuellini.

The genus *Shirozuella* is something of a taxonomic enigma. It is similar to Telsimiini of the Chilocorinae in having the *Chilocorus*-like clypeus, but the features of the antennae and maxillae, and the number of abdominal ventrites, as well as the structure of the head capsule are different than in the latter. A relatively close affinity with Sticholotidini is suggested by the very elongate maxillary palpi, the femora not strongly flattened, the relatively long antennae with a distinctly spindle shaped club, and the ventrally articulated labial palpi. Similarly, an affinity with Serangiini is supported by the possession of longitudinal furrows on the underside of the head, and by the nearly triangular median area of the prosternum. Based on this body of evidence, Sasaji (1967, 1968) placed *Shirozuella* in the tribe Shirozuellini of the subfamily Sticholotidinae (Sasaji 1967; 1968). Seago et al’s (2011) molecular analysis didn’t support Sasaji’s result, although it also failed to place Shirozuellini in any reasonable group. Instead, it adopted a two subfamily system for the family Coccinellidae as suggested by Ślipiński (2007), Microweiseinae and Coccinellinae, and placed Shirozuellini in Coccinellinae.

At present, the genus *Shirozuella* includes eight species distributed in Southeast and East Asia. Yu and Pang (1992) described the male genitalia of *Shirozuella mirabilis* and added two new species from Taiwan, *Shirozuella appendiculata* and *Shirozuella alishanensis*. Canepari and Milanese (1997) described a new species, *Ghanius schawalleri*, from Nepal Himalayas which was subsequently transferred to *Shirozuella* by Yu (2000[1999]). Four species were added from the Chinese mainland, *Shirozuella parenthesis*, *Shirozuella bimaculata*, *Shirozuella quadrimacularis*, *Shirozuella nibagou* (Yu 2000[1999]; Yu et al. 2000). The male genitalia of *Shirozuella bimaculata* were described by Ren et al. (2009).

Specimens of *Shirozuella* are rarely collected, and both sexes are known only for two of the eight previously described species. Most species were described based on only one or two specimens. In this paper, species of *Shirozuella* from the Chinese mainland are reviewed, and four species new to science are described, bringing the total to eight mainland species, and twelve species in all. Male genitalia of *Shirozuella parenthesis* and *Shirozuella quadrimacularis* are illustrated and described for the first time.

## Materials and methods

The specimens examined were collected from China. All of them were preserved in 85% ethanol. External morphology was observed with a dissecting stereo microscope (SteREO Discovery V20, Zeiss). The following measurements were made with an ocular micrometer: total length, from apical margin of clypeus to apex of elytra (TL); total width, across both elytra at widest part (TW=EW); height, through the highest point of elytra to metaventrite (TH); head width, including eyes (HW); pronotal length, from the middle of anterior margin to the base of pronotum (PL); pronotal width at widest part (PW); elytral length, along the suture, from the apex to the base including the scutellum (EL). Male and female genitalia were dissected, cleared in a 10% solution of NaOH by boiling for several minutes, and examined with an Olympus BX51 compound microscope.

Images were photographed with digital cameras (AxioCam HRc and Coolsnap–Pro*cf* & CRI Micro*Color), connected to the dissecting microscope. The software AxioVision Rel. 4.8 and Image–Pro Plus 5.1 were used to capture images from both cameras, and photos were cleaned up and laid out in plates with Adobe Photoshop CS 8.0.

Terminology follows Ślipiński (2007) and Ślipiński and Tomaszewska (2010).

The specimens are deposited in: South China Agriculture University, Guangzhou (SCAU), Institute of Plant & Environmental Protection, Beijing Academy of Agricultural & Forestry Science, Beijing (BAAF) and in the Institute of Zoology, Chinese Academy of Sciences, Beijing (IOZ).

## Taxonomy

### Genus Shirozuella

Sasaji, 1967

http://species-id.net/wiki/Shirozuella

Shirozuella Sasaji, 1967: 24. Type species: *Shirozuella mirabilis* Sasaji, 1967
Shirozuella : Yu and Pang 1992: 37; Miyatake 1994: 274; Yu et al. 2000: 187.

#### Diagnosis.

The tribe Shirozuellini includes five genera sharing many similar morphological characters: laterally expanded clypeus, very elongate terminal maxillary palpomere. *Shirozuella*, however can be distinguished from others by having: prosternum with distinct grooves anteriorly to receive antennae (Fig. 2) and maxillary palpi when the head is retracted; underside of head capsule with distinct grooves for reception of the retracted antennae; and antennae composed of nine antennomeres (Fig. 3).

#### Description.

**Figures 1–11.**
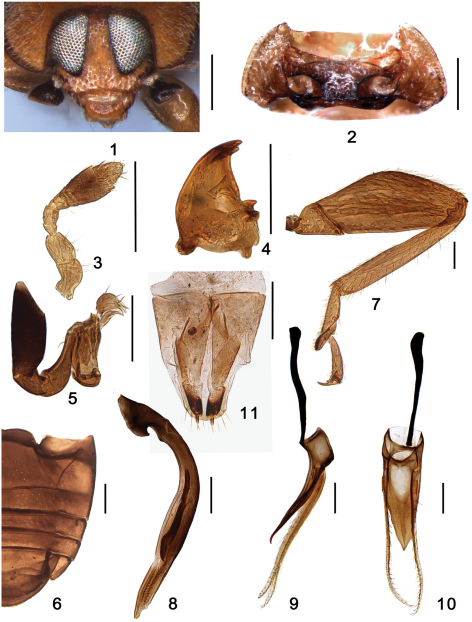
*Shirozuella bimaculata* Yu. **1** head, frontal view **2** prothorax, ventral **3** antenna **4** mandible **5** maxilla **6** abdomen **7** hind leg **8–10** male genitalia **8** penis **9** tegmen, lateral view **10** tegmen, ventral view **11** female genitalia: ovipositor. Scale bars: **1–2**: 0.2mm; **3–10**: 0.1mm.

**Figures 12–20.**
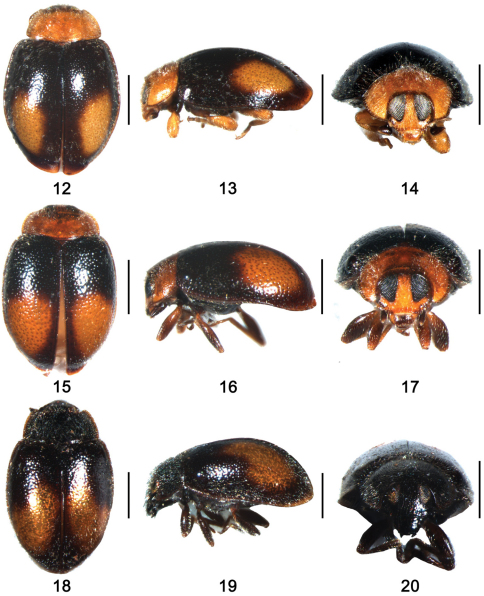
**12–14** *Shirozuella bimaculata* Yu. **12** dorsal view; **13** lateral view **14** frontal view **15–17** *Shirozuella motuoensis* sp. n. **15** dorsal view **16** lateral view **17** frontal view **18–20** *Shirozuella nibagou* Yu.**18** dorsal view **19** lateral view **20** frontal view. Scale bars: 0.5mm.

**Figures 21–29.**
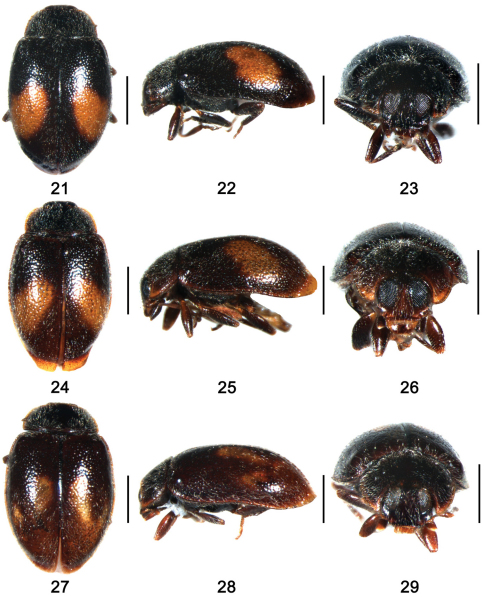
**21–23** *Shirozuella tibetina* sp. n. **21** dorsal view; **22** lateral view **23** frontal view **24–26** *Shirozuella unciforma* sp. n. **24** dorsal view **25** lateral view **26** frontal view **27–29** *Shirozuella quadrimacularis* Yu**27** dorsal view **28** lateral view **29** frontal view. Scale bars: 0.5mm.

Body small, oval, relatively elongate; dorsum weakly convex and pubescent (Figs 12–29). Head moderate in size, frontal surface of capsule flattened, slightly convex; clypeus long and distinctly expanded laterally; antennae inserted laterally near the apical end of head capsule; eyes large (Fig. 1). Antennae relatively long, composed of nine antennomeres with a distinct club composed of three antennomeres, eighth antennomere longest and widest, terminal antennomere rather small and conical (Fig. 3). Labrum relatively long. Mandible, bidentate at apex (Fig. 4). Maxillary palp much longer than antenna, composed of four palpomeres; terminal palpomere very elongate, and spatulate, more than twice as long as wide, with apex obliquely truncate (Fig. 5). Labial palp composed of 3 palpomeres and distal two palpomeres elongate and nearly cylindrical. Ventral surface of head capsule with distinct grooves for reception of retracted antennae.

Pronotum transverse, about twice as wide as long, strongly convex, lateral margins narrowly marginated and distinctly emarginated anteriorly. Scutellum medium-sized, triangular. Elytra elongate and weakly convex, with conspicuous swelling near humeral edge. Elytral base hardly wider than base of pronotum; elytral margins with very narrow rim, but distinctly visible from above. Hind wing well developed, with veins rather well-developed.

Prosternum short in front of coxae, distinctly grooved to receive antennae and palpi; prosternal process broad without longitudinal carinae. Mesoventrite broadly and nearly quadrate, slightly wider than long, anterior margin emarginate. Metaventrite broad, discrimen complete. Elytral epipleuron relatively narrow, nearly horizontal, not foveate, obsolete in apical third. Abdomen with six ventrites, ventrite 1 with complete postcoxal lines (Fig. 6). Legs relatively long; femora distinctly swollen and grooved to receive tibiae when retracted; tibiae slender and simple, tarsi composed of 3 tarsomeres (Fig. 7).

#### Distribution.

China (Gansu, Hunan, Henan, Shanxi, Shaanxi, Sichuan, Taiwan, Tibet, Yunnan), Japan, Nepal.

#### Key to species of *Shirozuella* Sasaji from the Chinese mainland

**Table:** 

1	Elytron with a yellow or yellowish brown spot or stripe	2
–	Elytron with two yellow or yellowish brown spots or stripes	6
2	Elytron with a nearly rounded spot	3
–	Elytron with a long and diagonal stripe	4
3	Penis short and stout, penis guide in ventral view nearly triangular, apex pointed (Fig. 10). TL: 1.71–1.86mm, TW: 1.19–1.32mm	*Shirozuella bimaculata*
–	Penis long and slender, penis guide in ventral view almost parallel at basal 2/3, apex truncate with a pair of small triangular projections on each side (Fig. 39). TL: 1.58–1.81mm, TW: 1.06–1.22 mm	*Shirozuella motuoensis*
4	Elytral macula emarginate antero-laterally (Figs 18–19). Abdominal postcoxal line complete, reaching ½ length of ventrite 1 (Fig. 41). TL: 1.88mm, TW: 1.19 mm	*Shirozuella nibagou*
–	Elytral macula without antero-lateral emargination Abdominal postcoxal line complete, reaching more than ½ length of ventrite 1	*5*
5	Elytra black, apex very narrowly brown (Figs 21–22). Anterior angles of pronotum black (Fig. 23). Apex of penis guide pointed, slightly curved outwardly (Fig. 46). TL: 1.68–1.75mm, TW: 1.02–1.12mm	*Shirozuella tibetina*
–	Elytra reddish brown, apical 1/10 yellow (Figs 24–25). Anterior angles of pronotum yellow (Fig. 26). Apex of penis guide unciform (Fig. 51). TL: 1.65–1.75mm, TW: 1.12–1.29mm	*Shirozuella unciforma*
6	Each elytron with two longitudinal yellow spots (Fig. 27). Pronotum black (Fig. 27). TL: 1.86mm, TW: 1.2mm	*Shirozuella quadrimacularis*
–	Each elytron with two transverse bands. Pronotum yellow, or bicolored	7
7	Pronotum with disk black and lateral margins yellow (Fig. 30). TL: 1.81–2.31mm, TW: 1.24–1.58mm	*Shirozuella parenthesis*
–	Pronotum yellow (Fig. 33). TL: 1.94mm, TW: 1.32mm	*Shirozuella guoyuei*

### Shirozuella bimaculata

Yu, 2000

http://species-id.net/wiki/Shirozuella_bimaculata

Figs 1 –14 68

Shirozuella bimaculata Yu, 2000[1999]: 63; Ren et al. 2009: 42.

#### Diagnosis.

This species can be identified as follows: pronotum uniformly yellow (Fig. 12), elytron black with a large yellow spot (Fig. 12), penis short and stout (Fig. 8), and penis guide short and subtriangular (Figs 9–10).

#### Description.

TL: 1.71–1.86mm, TW: 1.19–1.32mm, TH: 0.63–0.69mm, TL/TW: 1.41–1.44; PL/PW: 0.44–0.47; EL/EW: 1.08–1.14.

Body small, elongate oval, weakly convex, dorsum covered with relatively sparse pubescence (Figs 12–14). Head yellow with maxillary palpus dark brown. Pronotum yellow, scutellum dark brown. Elytra blackish, with two large yellow spots placed on apical half, apex narrowly yellow. Ventral surfaces uniformly dark brown, legs yellow.

Head moderately large (Fig. 1), 0.35× elytral width (HW/EW=2.86); frontal surface of head capsule slightly convex; punctures on frons fine and sparse, separated by about 2.0–3.0× a diameter, with sparse long setae in punctures; eyes large, narrowly separated; widest interocular distance 2× narrowest width. Pronotum relatively small, 0.74× elytral width (PW/EW=1:1.36), pronotal punctures fine, inconspicuous, smaller than those on head, separated by 3.0–4.0× a diameter. Scutellum moderately large, triangular, visible from above. Punctures on elytra moderately large, irregular, obviously larger than those on pronotum, separated by 1.0–2.0× a diameter.

Surface of prosternum slightly shagreened, punctures inconspicuous, with sparse short setae. Mesoventrite shiny and glabrous, punctures inconspicuous, with several short setae. Metaventrite broad and glabrous, median part concave, with complete median discrimen; punctures sparse and large, with short sparse setae. Abdominal postcoxal line complete, v-shaped, reaching to 2/3 length of ventrite 1, or almost touching posterior margin (Fig. 6).

Male genitalia: Penis short and stout, penis capsule with indistinct outer arm and short inner one (Fig. 8); penis guide in lateral view slender, widest at base, tapering to apex, apex pointed and slightly curved (Fig. 9); parameres slender, sparsely setose on apical half, about 1.65× as long as penis guide (Fig. 9); penis guide in ventral view short and stout, subtriangular, widest at base, tapering to apex, apex pointed (Fig. 10).

Female genitalia: Coxites elongate, about 3.0 times as long as wide, tapering to blunt, darker apices, styli distinct, with short terminal setae (Fig. 11); spermatheca not sclerotized.

#### Types examined.

**Holotype:** 1♀, **China, Henan:** Huangshian, Laojieling, Taiping Town, Xixia County, [33°37.53'N, 111°46.17'E], ca 1500m, 19.vii.1998, Yu Guoyue leg. (BAAF).

#### Other specimens examined.

1♂1♀, **China, Gansu:** Maijishan Forest Park, Tianshui, [34°20.81'N, 106°0.71'E], ca 1500m, 10.viii.2008, Wang XM leg. (SCAU); 4♂♂, Maijishan Forest Park, Tianshui, [34°20.81'N, 106°0.71'E], ca 1650m, 6.viii.2009, Wang XM et al. leg. (3♂♂ SCAU; 1♂ IOZ); **Henan:** 2♂♂2♀♀, Longyuwan Forest Park, Luanchuan, [33°41.16'N, 111°50.72'E], ca 1400m, 11.vii.2009, Wang XM et al. Leg. (1♂1♀ SCAU, 1♂1♀ IOZ); **Shaanxi:** 2♂♂2♀♀, Xunyangba, Ningshan, [33°32.87'N, 108°33.13'E], ca 1600m, 23.vii.2009, Wang XM et al. Leg. (SCAU).

#### Distribution.

China (Gansu, Henan, Shaanxi).

**Figures 30–35.**
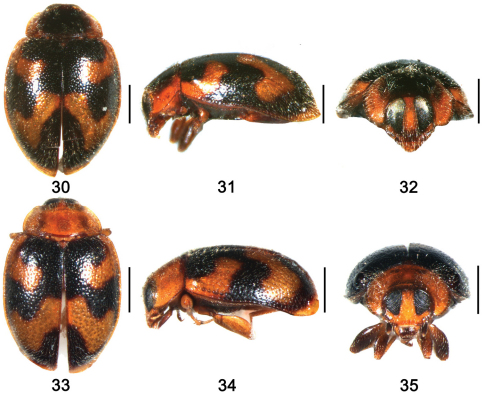
**30–32** *Shirozuella parenthesis* Yu **30** dorsal view **31** lateral view **32** frontal view **33–35** *Shirozuella guoyuei* sp. n. **33** dorsal view **34** lateral view **35** frontal view.Scale bars: 0.5mm.

### Shirozuella motuoensis sp. n.

urn:lsid:zoobank.org:act:8705EDF0-F6A7-4298-94A6-8A9F2AB90F4F

http://species-id.net/wiki/Shirozuella_motuoensis

Figs 15–17 36–40 68

#### Diagnosis.

This species is close to *Shirozuella bimaculata* in general appearance, but it is easily distinguished from the latter by the long and slender penis and the special shape of penis guide (Figs 37–39).

#### Description.

TL: 1.58–1.81mm, TW: 1.06–1.22mm, TH: 0.63–0.82mm, TL/TW: 1.49–1.50; PL/PW: 0.48–0.50; EL/EW: 1.14–1.31.

Body small, elongate oval, weakly convex, dorsum covered with relatively sparse pubescence (Figs 15–17). Head yellow to yellowish brown, with labrum and maxillary palpus brown. Pronotum yellowish brown, scutellum dark brown. Elytra blackish, with two large yellow spots placed on apical half, apex narrowly yellow. Prosternum dark brown, meso- and metaventrite black, elytral epipleura and legs brown.

Head moderately large, 0.41× elytral width (HW/EW=2.46); frontal surface of head capsule slightly convex and rather weakly anteriorly inclined below; punctures on frons fine and sparse, inconspicuous, separated by 2.0–4.0× a diameter, with sparse long setae in punctures; eyes relatively large, narrowly separated; widest interocular distance 1.3× narrowest width. Pronotum relatively small, 0.69× elytral width (PW/EW=1: 1.45), pronotal punctures very fine and inconspicuous, smaller than those on head, separated by 3.0–4.0× a diameter. Scutellum moderately large, triangular, visible from above. Punctures on elytra moderately large, irregular, obviously larger than those on pronotum, separated by 1.0–2.5× a diameter.

**Figures 36–43.**
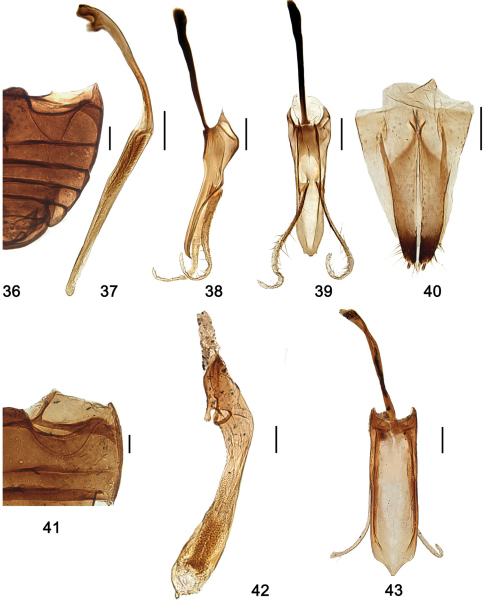
**36–40** *Shirozuella motuoensis* sp. n. **36** abdomen **37–39** male genitalia **37** penis **38** tegmen, lateral view **39** tegmen, ventral view **40** female genitalia: ovipositor **41–43** *Shirozuella nibagou* Yu **41** abdomen **42–43** male genitalia **42** penis **43** tegmen, ventral view. Scale bars: 0.1 mm.

Surface of prosternum mat and slightly shagreened, punctures inconspicuous, with sparse short setae. Mesoventrite slightly shagreened, punctures inconspicuous, with several short setae. Metaventrite broad and glabrous, median part concave, with complete median discrimen; punctures sparse and fine, separated by 2.0–6.0× a diameter, with short sparse setae. Abdominal postcoxal line complete, v-shaped, touching posterior margin of ventrite 1 (Fig. 36).

Male genitalia: Penis long and slender, strongly curved at 2/5 length, distinctly swollen at middle, penis capsule small (Fig. 37); penis guide in lateral view slender, widest at base, tapering to apex, apex pointed and slightly curved (Fig. 38); parameres slender, sparsely setose on apical half, about 2× as long as penis guide, always strongly curved at apical 2/5 length (Fig. 38); penis guide in ventral view short and stout, almost parallel at basal 2/3, then tapering to apex, apex truncated with a pair of small triangular projections on each side (Fig. 39).

Female genitalia: Coxities elongate, about 3.5× as long as wide, tapering to blunt, darker apices, styli small and distinct, with short terminal setae, apical area distinctly darkened (Fig. 40); spermatheca not sclerotized.

#### Types.

**Holotype:** 1♂, **China, Tibet:** Hanmi, Motuo County, [29°°21.87'N, 95°7.75'E], ca 2100m, 13.x.2009, Wang XM leg. (SCAU). **Paratypes: China, Tibet:** 2♀♀, same data as holotype; 1♂, Yarang village, Motuo, [29°17.70'N, 95°16.87'E], ca 1000m, 24.x.2007, Wang XM leg. (SCAU).

#### Distribution.

China (Tibet).

#### Etymology.

The specific epithet refers to Motuo, the type locality of this ladybird.

### Shirozuella nibagou

Yu, 2000

http://species-id.net/wiki/Shirozuella_nibagou

Figs 18–20 41–43 68

Shirozuella nibagou : Yu et al., 2000: 188.

#### Diagnosis.

This species is easily distinguished from other *Shirozuella* by black body and elytron with a yellowish curved spot (Figs 18–19).

#### Description.

TL: 1.88mm, TW: 1.19mm, TH: 0.76mm, TL/TW: 1.58; PL/PW: 0.56; EL/EW: 1.03.

Body small, elongate oval, weakly convex. Dorsum covered with relatively sparse pubescence (Figs 18–20). Head black. Pronotum and scutellum black. Elytron blackish, with a curved large yellowish brown spot situated between 2/5 and 4/5 elytral length to apex. Underside uniformly dark.

Head moderately large, 0.36× elytral width (HW/EW=2.77); frontal surface of head capsule flattened and rather weakly anteriorly inclined below; punctures on frons fine and sparse, separated by 2.0–3.0× a diameter, with sparse long setae in punctures; eyes relatively large, narrowly separated; widest interocular distance 1.3× narrowest width. Pronotum 0.69× elytral width (PW/EW=1: 1.44), pronotal punctures very fine, inconspicuous, smaller than those on head. Scutellum moderately large, triangular, visible from above. Punctures on elytra large, irregular, obviously larger than those on head, separated by 0.2–0.5× a diameter.

Surface of prosternum slightly mat and shagreened, punctures inconspicuous, with sparse short setae. Mesoventrite shiny and glabrous, punctures inconspicuous, with several short setae. Metaventrite broad and glabrous, median part concave, with complete median discrimen; punctures sparse and fine, with short sparse setae. Abdominal postcoxal line complete, reaching to 1/2 length of ventrite 1, then recurved basally (Fig. 41).

Male genitalia: Penis short and stout, penis capsule indistinct, apex rounded by membrane (Fig. 42); penis guide in lateral view slender, widest at base, tapering to apex, apex pointed and slightly curved; parameres slender, sparsely setose; penis guide in ventral view short and stout, parallel at basal 6/7, then converging sharply to teat-like tip (Fig. 43).

Female genitalia: Coxities elongate, tapering to blunt, darker apices, styli small and distinct, with short terminal setae; spermatheca not sclerotized.

#### Types examined.

**Holotype:** 1♂, **China, Sichuan:** Nibagou, Baoxing, [30°41.31'N, 102°42.87'E], ca viii.1997, Guo H. leg. (BAAF). **Paratype:** 1♀, same data as holotype.

#### Distribution.

China (Sichuan).

### Shirozuella tibetina sp. n.

urn:lsid:zoobank.org:act:BE699A24-2054-47FD-B1F2-D744ABBD67D0

http://species-id.net/wiki/Shirozuella_tibetina

Figs 21–23 44–48 68

#### Diagnosis.

**Figures 44–53.**
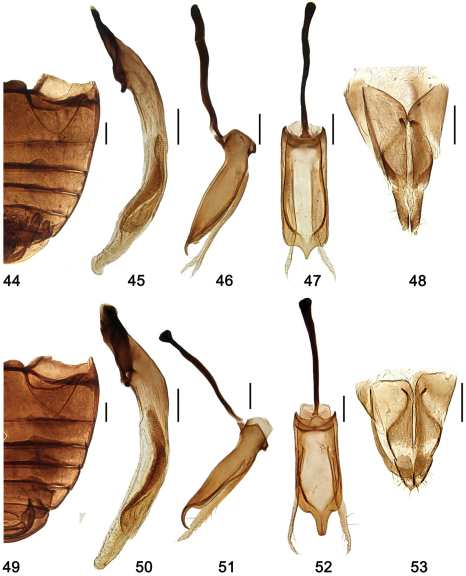
**44–48** *Shirozuella tibetina* sp. n. **44** abdomen **45–47** male genitalia **45** penis **46** tegmen, lateral view **47** tegmen, ventral view **48** female genitalia: ovipositor. **49–53** *Shirozuella unciforma* sp. n. **49** abdomen; **50–52** male genitalia: **50** penis **51** tegmen, lateral view **52** tegmen, ventral view **53** female genitalia: ovipositor. Scale bars: 0.1 mm.

This species is close to *Shirozuella nibagou* in general appearance, but it can be distinguished from the latter as follows: posterior margin of abdominal postcoxal line reaching to 4/5 ventrite length (Fig. 44), and elytral spots are not curved (Figs 21–22). In *Shirozuella nibagou*, posterior margin of abdominal postcoxal line reaching to 1/2 ventrite length (Fig. 41), and elytral spots are curved (Figs 18–19). The male genitalia are also diagnostic.

This species is also similar to *Shirozuella schawalleri* (Canepari & Milanese, 1997) in general appearance, but it can be distinguished from the latter by elytra with a curved yellowish brown spot and elongated triangular coxities. In *Shirozuella schawalleri*, spots on elytra are straight and oblique and outer margins of coxities arcuate.

#### Description.

TL: 1.68–1.75mm, TW: 1.02–1.12mm, TH: 0.66–0.76mm, TL/TW: 1.41–1.44; PL/PW: 0.51–0.52; EL/EW: 1.18–1.19.

Body small, elongate oval, weakly convex, dorsum covered with sparse pubescence (Figs 21–23). Head black with maxillary palpus brown. Pronotum black with anterior slightly brown, scutellum black. Elytron black, with a curved yellowish brown spot situated between 2/5 and 4/5 elytral length to apex. Ventral surface dark, except elytral epipleura and legs brown.

Head moderately large, 0.38× elytral width (HW/EW=2.62); frontal surface of head capsule flattened and rather weakly anteriorly inclined below; punctures on frons fine, separated by 2.0–4.0× a diameter, with sparse long setae in punctures; eyes relatively large, narrowly separated; widest interocular distance about 1.5× narrowest width. Pronotum 0.68× elytral width (PW/EW=1: 1.48), pronotal punctures very fine, smaller than those on head, separated by 2.0–4.0× a diameter. Scutellum moderately large, triangular. Punctures on elytra moderately large, obviously larger than those on pronotum, separated by 1.5–2.5× a diameter.

Pro- and mesoventrite slightly shagreened, punctures inconspicuous, with sparse short setae. Metaventrite broad and glabrous, median part concave, with complete median discrimen; punctures fine and sparse, separated by about 2.0–4.0× a diameter, with short sparse setae. Abdominal postcoxal line complete, reaching to 4/5 length of ventrite 1 (Fig. 44).

Male genitalia: Penis short and stout, penis capsule distinct, apex blunt and slightly swollen (Fig. 45); penis guide in lateral view stout, widest at basal 2/5, apex pointed and curved (Fig. 46); parameres slender, sparsely setose at apex, distinctly longer than penis guide (Fig. 46); penis guide in ventral view short and stout, parallel at basal 10/11, then converging sharply to pointed apex (Fig. 47).

Female genitalia: Coxities elongate, subtriangular, about 3.5 × as long as wide, tapering to blunt, darker apices, styli small and distinct, with short terminal setae (Fig. 48); spermatheca not sclerotized.

#### Types.

**Holotype: China, Tibet:** 1♂, Xiayadong town, Yadong, [28°29.29'N, 97°1.36'E], ca 2800m, 1.x.2009, Wang XM et al. leg.(SCAU). **Paratypes: Tibet:** 1♂6♀♀, same data as holotype (SCAU); 2♀♀, North of Chayu City, [28°42.03'N, 97°27.77'E], ca 2300m, 18.x.2007, Wang XM leg. (SCAU); 1♂1♀, Le Village, Cuona, [27°48.63'N, 91°44.98'E], ca 2400m, 3.x.2009, Wang XM et al. Leg. (SCAU).

#### Distribution.

China (Tibet).

#### Etymology.

The specific epithet is in reference to Tibet, the type locality of this ladybird.

### Shirozuella unciforma sp. n.

urn:lsid:zoobank.org:act:21E76C93-6EB3-4933-820B-46F86E2E142F

http://species-id.net/wiki/Shirozuella_unciforma

Figs 24–26 49–53 68

#### Diagnosis.

This species is close to *Shirozuella tibetina*, but it can be distinguished from the latter as follows: elytral apex is distinct yellow (Figs 24–25), penis apex is slightly swollen and penis guide’s apex unciform (Figs 50–51). Coxities are also diagnostic (Fig. 53).

#### Description. 

TL: 1.65–1.75mm, TW: 1.12–1.29mm, TH: 0.66–0.73mm, TL/TW: 1.36–1.47; PL/PW: 0.52–0.55; EL/EW: 1.05–1.12.

Body small, elongate oval, weakly convex, dorsum covered with relatively sparse pubescence (Figs 24–26). Head brown to black. Pronotum black with anterior corners clearly yellowish brown, scutellum black. Elytron brown to black, with an elongate oblique yellowish brown spot situated between 2/5 and 4/5 elytral length to apex. Pro- and mesoventrite brown, metaventrite black, elytral epipleura brown. Legs brown with coxae yellow.

Head moderately large (Fig. 26), about 0.31× elytral width (HW/EW=3.25); frontal surface of head capsule slightly convex and rather weakly anteriorly inclined below; punctures on frons fine, separated by 2.0–2.5× a diameter, with sparse long setae in punctures; eyes relatively large, narrowly separated; widest interocular distance about 1.5× narrowest width. Pronotum relatively small, 0.62× elytral width (PW/EW=1: 1.63), pronotal punctures very fine, smaller than those on head, separated by 1.5–3.5× a diameter. Scutellum moderately large, triangular, visible from above. Elytra rather elongate and weakly convex, with conspicuous swelling near humeral edge. Punctures on elytra moderately large, irregular, obviously larger than those on pronotum, separated by 1.5–2.5× a diameter.

Pro- and mesoventrite slightly shagreened, punctures inconspicuous, with several short setae. Metaventrite broad and glabrous, median part concave, with complete median discrimen; punctures sparse and fine, separated by 2.5–3.0× a diameter, with short sparse setae. Abdominal postcoxal line complete, reaching to 4/5 length of ventrite 1 (Fig. 49).

Male genitalia: Penis short and stout, penis capsule inconspicuous, apex rounded (Fig. 50); penis guide in lateral view stout, widest at base, gradually tapering to 4/5 length, then abruptly narrowed, apex unciform (Fig. 51); parameres slender, straight, sparsely setose at basal half, distinctly longer than penis guide; penis guide in ventral view short and stout, almost parallel at basal 4/5, then converging sharply to apex, apex slightly blunt (Fig. 52).

Female genitalia: Coxities elongate, subtriangular, about 2.5× as long as wide, tapering to blunt apices, styli very small and inconspicuous, each with short terminal setae (Fig. 53); spermatheca not sclerotized.

#### Types.

**Holotype:** 1♂, **China. Tibet:** Hanmi, Motuo County, [29°21.87'N, 95°7.75'E], ca 2100m, 13.x.2009, Wang XM leg. (SCAU). **Paratype**: 1♀, same data as holotype.

#### Distribution.

China (Tibet).

#### Etymology.

The specific epithet is formed from the Latin adjective *unciformus*, referring to unciform apex of penis guide.

### Shirozuella quadrimacularis

Yu, 2000

http://species-id.net/wiki/Shirozuella_quadrimacularis

Figs 27–29 54–58 68

Shirozuella quadrimacularis : Yu et al., 2000: 187; Ren et al. 2009: 42.

#### Diagnosis.

This species can be distinguished from other *Shirozuella* by elytra black with four longitudinal yellow spots (Figs 27–28).

#### Description.

TL: 1.86mm, TW: 1.2mm, TH: 0.71mm, TL/TW: 1.55; PL/PW: 0.42; EL/EW: 1.25.

Body small, elongate oval, weakly convex. Dorsum covered with relatively sparse pubescence (Figs 27–28). Head black with mouth parts brown. Pronotum black, with lateral margins and anterior corners brown. Scutellum black. Elytron blackish, with two longitudinal yellow spots, one situated at middle of elytral length, less than its width from suture, the other spot smaller, situated slightly past middle, distance to lateral margin slightly more than width of spot, elytral apex slightly yellow. Ventral surfaces uniformly black, except elytral epipleura brown. Legs brown with coxae and tarsi yellow.

Head moderately large (Fig. 29), 0.40× elytral width (HW/EW=2.50); frontal surface of head capsule slightly convex and rather weakly anteriorly inclined below; punctures on frons fine and inconspicuous, with sparse long setae in punctures; eyes relatively large, narrowly separated; widest interocular distance about 1.5× narrowest width. Pronotum 0.68× elytral width (PW/EW=1: 1.48), pronotal punctures extremely fine, separated by 3.0–5.0× a diameter. Scutellum moderately large, triangular. Punctures on elytra moderately large, irregular, obviously larger than those on pronotum, separated by 1.0–2.0× a diameter.

Pro- and mesoventrite slightly shagreened, punctures inconspicuous, with sparse short setae. Metaventrite broad and glabrous, median part concave, with complete median discrimen; punctures sparse and fine, with short sparse setae. Abdominal postcoxal line complete, v-shaped, touching 2/3 length of ventrite 1 (Fig. 45).

Male genitalia: Penis short and stout, penis capsule small, apex rounded by membrane (Fig. 55); penis guide in lateral view stout, gradually narrowing to apex, apex pointed and curved (Fig. 56); parameres slender, sparsely setose at apex, distinctly longer than penis guide (Fig. 56); penis guide in ventral view short and stout, parallel at basal 10/11, then converging sharply to pointed apex (Fig. 57).

Female genitalia: Coxities elongate, about 3.5× as long as wide, tapering to blunt apices, styli small and distinct, with short terminal setae (Fig. 58); spermatheca not sclerotized.

#### Types examined.

**Holotype: China. Yunnan:** 1♀, Lijiang, iv.1996, Yu GY leg. (BAAF).

#### Other specimens examined.

**China, Sichuan:** 1♀, Dafengding, Meigu, [28°31.03'N, 103°18.22'E], ca 2400m, 21.ix.2007, Wang XM leg. (SCAU); 1♂1♀, Pass 30km SW Mianning [28°27.87'N, 101°58.51'E], ca 3000–3400m, 11–13.VII.2007, S. Murzin leg. (SCAU).

#### Distribution.

China (Sichuan, Yunnan).

### Shirozuella parenthesis

Yu, 2000

http://species-id.net/wiki/Shirozuella_parenthesis

Figs 30–32 59–63 68

Shirozuella parenthesis Yu, 2000[1999]: 62.

#### Diagnosis. 

This species can be identified by pronotum yellowish brown, with a large quadrate black spot and black elytron with two large curved yellowish brown strips (Figs 30–31).

#### Description.

TL: 1.81–2.31mm, TW: 1.24–1.58mm, TH: 0.82–0.89mm, TL/TW: 1.46–1.47; PL/PW: 0.51–0.52; EL/EW: 1.10–1.15.

Body small, elongate oval, weakly convex. Dorsum covered with relatively sparse pubescence (Figs 30–31). Head brown, with maxillary palpus dark brown. Pronotum yellowish brown, with a large quadrate black spot. Scutellum dark brown. Elytron black, with two large curved yellowish brown strips, one C-shaped, situated around out margin of humeral callus, connected to basal margin of elytron, another one v-shaped, situated between 2/5 and 4/5 elytral length to apex; apex of elytron narrowly yellowish brown. Ventral surfaces uniformly dark brown, except elytral epipleura and legs brown.

Head moderately large (Fig. 32), 0.37× elytral width (HW/EW=2.68); frontal surface of head capsule flatten and rather weakly anteriorly inclined below; punctures on frons fine and dense, separated by 1.5–2.0× a diameter, with sparse long setae in punctures; eyes relatively large, distinctly broader than interocular distance; widest interocular distance about 1.6× narrowest width. Pronotum relatively small, 0.67× elytral width (PW/EW=1: 1.50), pronotal punctures very fine, smaller than those on head, separated by 2.0–5.0× a diameter. Scutellum moderately large, triangular. Punctures on elytra moderately large, obviously larger than those on pronotum, separated by 1.0–3.0× a diameter.

Pro- and mesoventrite slightly shagreened, punctures inconspicuous, with sparse short setae. Metaventrite broad and glabrous, median part flatten, with complete median discrimen; punctures on lateral area moderated large, separated by about 1.5–3.0× a diameter, with short sparse setae. Abdominal postcoxal line complete, reaching to 2/3 length of ventrite 1, then recurved basally (Fig. 59).

Male genitalia: Penis long and slender, penis capsule inconspicuous (Fig. 60); penis guide in lateral view slender, widest at base, tapering to apex, apex pointed and curved (Fig. 61); parameres slender, sparsely setose at apical half, longer than penis guide, apex curved (Fig. 61); penis guide in ventral view almost parallel at basal 3/4, then gradually narrowing to apex, apex truncated (Fig. 62).

Female genitalia: Coxities elongate, subtriangular, about 5.3× as long as wide, tapering to blunt, darker apices, styli small and distinct, each with short terminal setae (Fig. 63); spermatheca not sclerotized.

#### Types examined.

**Holotype:** 1♀, **China, Henan:** Huangshian, Laojieling, Taiping Town, Xixia County, [33°37.53'N, 111°46.17'E], ca 1500m, 19.vii.1998, Yu GY leg. (BAAF).

#### Other specimens examined.

1♂2♀♀, **China, Shanxi:** Lishan National Natural Reserve, Yuanqu, [35°28.39'N, 112°9.45'E], ca 800–1400m, 3.viii.2011, Wang XM et al. leg. (SCAU).

#### Distribution.

China (Henan, Shanxi).

### Shirozuella guoyuei sp. n.

urn:lsid:zoobank.org:act:4A74A1CF-AAD1-4CD7-BFC9-0D275A23FB1A

http://species-id.net/wiki/Shirozuella_guoyuei

Figs 33–35 64- 68

#### Diagnosis.

This species is colse to *Shirozuella parenthesis* in general appearance, but it can be distinguished from the latter by the distance between eyes (Fig. 35), yellow pronotum (Fig. 33), stout penis and penis guide (Figs 65–67).

#### Description.

TL: 1.94mm, TW: 1.32mm, TH: 0.73mm, TL/TW: 1.48; PL/PW: 0.53; EL/EW: 1.15.

Body small, elongate oval, weakly convex. Dorsum covered with relatively sparse pubescence (Figs 33–34). Head brown, with terminal maxillary palpomere dark brown. Pronotum and scutellum yellow. Elytron black, with two large curved yellowish stripes, one c-shaped, situated around outer margin of humeral callus, connected to lateral margin of elytron, another one v-shaped, situated between 2/5 and 4/5 elytral length to apex, connected to elytral suture; apex of elytron yellow. Underside uniformly dark brown, except elytral epipleura and legs yellow.

Head moderately large (Fig. 35), 0.35× elytral width (HW/EW=2.86); frontal surface of head capsule slightly convex and rather weakly anteriorly inclined below; punctures on frons fine, separated by 0.5–1.5× a diameter, with sparse long setae in punctures; eyes relatively large, distinctly broader than interocular distance; widest interocular distance 3.2× narrowest width. Pronotum 0.66× elytral width (PW/EW=1: 1.51), pronotal punctures fine, smaller than those on head, separated by 1.0–3.0× a diameter. Scutellum moderately large, triangular. Punctures on elytra moderately large, irregular, obviously larger than those on pronotum, separated by 1.0–2.0× a diameter.

Pro- and mesoventrite mat and shagreened, punctures inconspicuous, with sparse short setae. Metaventrite broad and glabrous, median part concaved, with complete median discrimen; punctures sparse and fine, separated by 2.0–3.0× a diameter, with short sparse setae. Abdominal postcoxal line complete, v-shaped, nearly reaching to posterior margin of ventrite 1 (Fig. 64).

Male genitalia: Penis short and stout, slightly curved, without penis capsule (Fig. 65); penis guide in lateral view widest at base, gradually narrowed to apex, apex pointed and curved (Fig. 66); parameres slender, sparsely setose at apical half, longer than penis guide, apex curved (Fig. 66); penis guide in ventral view almost parallel at basal 6/7, then gradually narrowing to apex, apex truncated (Fig. 67).

Female genitalia: unknown.

#### Types.

**Holotype:** 1♂, **China, Hunan:** Tianpingshan, Zhangjiajie, [29°43.02'N, 109°49.14'E], ca 950m, 14.viii.2001, Peng ZQ leg. (SCAU).

#### Distribution.

China (Hunan).

#### Etymology.

The species is named after Dr. Yu Guoyue, an outstanding coccinellid taxonomist of China

**Figures 54–63.**
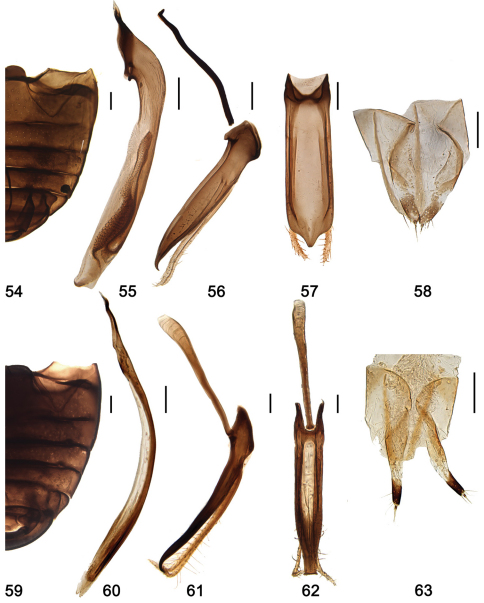
**54–58** *Shirozuella quadrimacularis* Yu **54** abdomen **55–57** male genitalia **55** penis **56** tegmen, lateral view **57** tegmen, ventral view **58** female genitalia: ovipositor **59–63** *Shirozuella parenthesis* Yu**59** abdomen **60–62** male genitalia **60** penis **61** tegmen, lateral view **62** tegmen, ventral view **63** female genitalia ovipositor. Scale bars: 0.1 mm.

**Figures 64–67.**
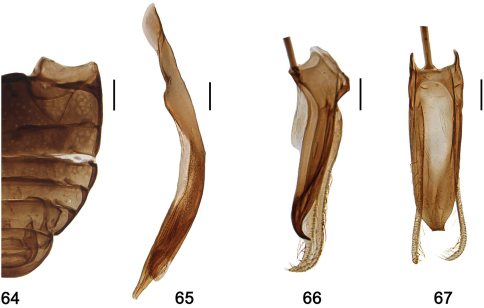
*Shirozuella guoyuei* sp. n. **64** abdomen **65–67** male genitalia **65** penis **66** tegmen, lateral view **67** tegmen, ventral view. Scale bars: 0.1 mm.

**Figure 68.**
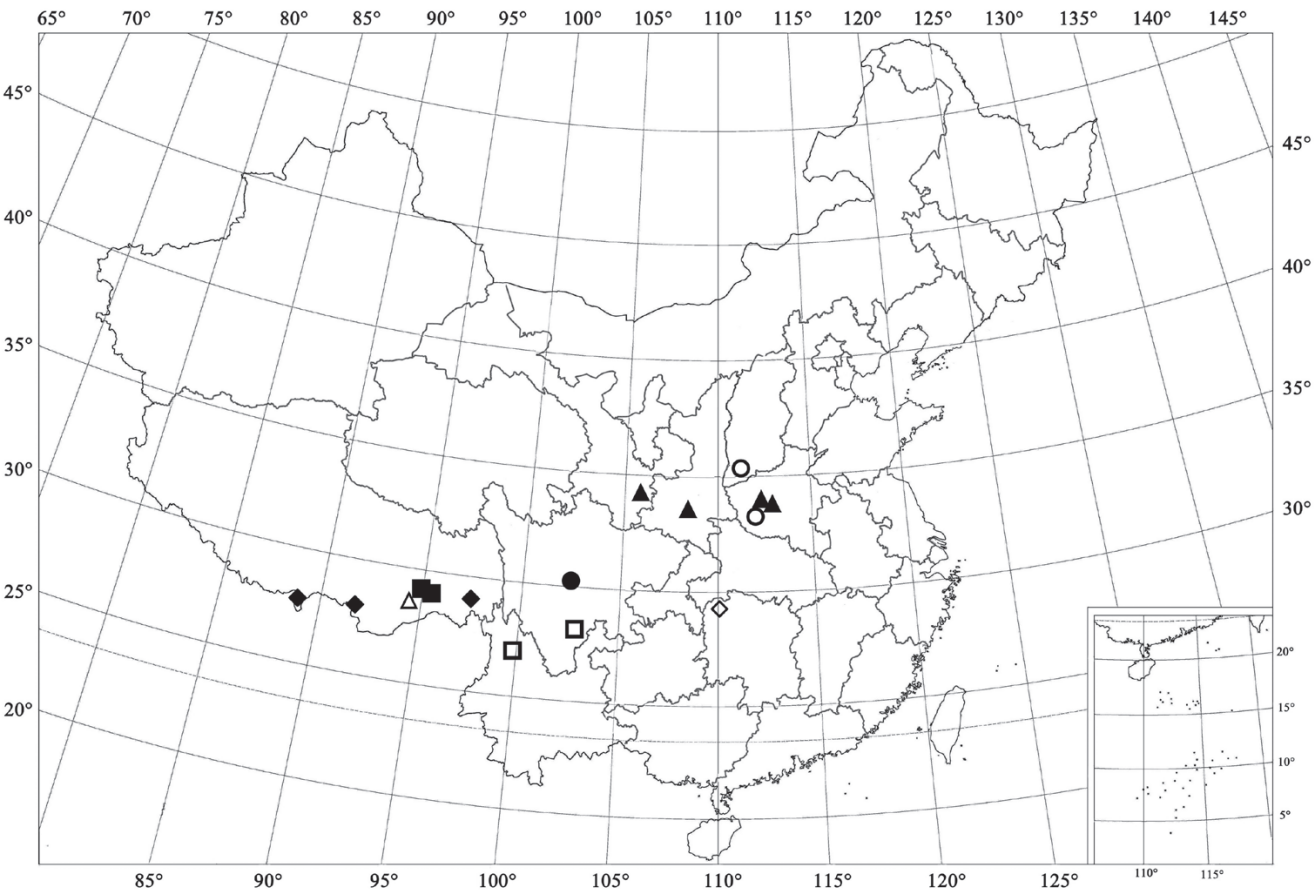
Distribution map. ▲ *Shirozuella bimaculata* Yu ■ *Shirozuella motuoensis* sp. n. ● *Shirozuella nibagou* Yu ◆ *Shirozuella tibetina* sp. n. △ *Shirozuella unciforma* sp. n. □ *Shirozuella quadrimacularis* Yu ○ *Shirozuella parenthesis* Yu ◊ *Shirozuella guoyuei* sp. n.

## Supplementary Material

XML Treatment for Shirozuella

XML Treatment for Shirozuella bimaculata

XML Treatment for Shirozuella motuoensis

XML Treatment for Shirozuella nibagou

XML Treatment for Shirozuella tibetina

XML Treatment for Shirozuella unciforma

XML Treatment for Shirozuella quadrimacularis

XML Treatment for Shirozuella parenthesis

XML Treatment for Shirozuella guoyuei
